# Calculating bond dissociation energies of X−H (X=C, N, O, S) bonds of aromatic systems via density functional theory: a detailed comparison of methods

**DOI:** 10.1098/rsos.220177

**Published:** 2022-06-08

**Authors:** Nguyen Quang Trung, Adam Mechler, Nguyen Thi Hoa, Quan V. Vo

**Affiliations:** ^1^ Department of Chemistry, The University of Danang - University of Science and Education, Da Nang 550000, Vietnam; ^2^ Quality Assurance and Testing Center 2, Danang 550000, Vietnam; ^3^ Department of Chemistry and Physics, La Trobe University, Victoria 3086, Australia; ^4^ Academic Affairs, The University of Danang - University of Technology and Education, Danang 550000, Vietnam; ^5^ Faculty of Chemical Technology – Environment, The University of Danang - University of Technology and Education, Danang 550000, Vietnam

**Keywords:** DFT, functional, bond dissociation energy, aromatic compounds, M06-2X, M08-HX

## Abstract

In this study, the performance of 17 different density functional theory functionals was compared for the calculation of the bond dissociation energy (BDE) values of X−H (X=C, N, O, S) bonds of aromatic compounds. The effect of the size of the basis set (expansions of 6-31(G)) was also assessed for the initial geometry and zero-point energy calculations, followed by the single-point BDE calculations with different model chemistries with the 6-311 + (3df,2p) basis set. It was found that the size of the basis set for geometry optimization has a much smaller effect on the accuracy of BDE than the choice of functional for the following single-point calculations. The M06-2X, M05-2X and M08−HX functionals yielded highly accurate BDE values compared to experimental data (with the average mean unsigned error MUE = 1.2–1.5 kcal mol^−1^), performing better than any of the other functionals. The results suggest that geometry optimization may be performed with B3LYP functional and a small basis set, whereas the M06-2X, M05-2X and M08-HX density functionals with a suitably large basis set offer the best method for calculating BDEs of ArX−H (X=C, N, O, S) bonds.

## 1. Introduction

The Kohn–Sham density functional theory (DFT) offers one of the most widely used methods in computational and theoretical chemistry, with significant progress achieved in developing and validating highly accurate exchange and correlation functionals during the last decade [1–4]. The broad range of model chemistries offers various strengths and weaknesses, where higher accuracy usually means increasingly, often prohibitively high cost in terms of CPU time. As the development effort reached the final frontiers within the limitations of the method, DFT has increasingly turned into a routine tool for practical applications [5–8]. With a parallel increase in desktop computing power, the DFT method now advanced from the realm of specialists to become a mainstream, routine method for any chemist seeking fast validation for reaction pathways and energetics or predictions of various physico-chemical properties of compounds. That necessitates moving away from the ‘higher is better’ attitude towards the level of theory and a kind of cost-benefit analysis: for practical purposes, what level of theory is sufficient to address a particular problem [9].

Bond dissociation energy (BDE) is defined as the enthalpy change of the homolytic cleavage of a specific bond, providing one of the essential characteristics of the breakdown reactivities of compounds in the context of e.g. their antioxidant activity [10,11]. Experimental methods to determine BDE are limited to measuring energy flows, such as calorimetry, and rely on breaking a known weak bond. Thus, experimental measurements of BDE are limited to simple and/or small molecules, hence they are unsuitable in many real-world situations where large and complicated molecules are involved, which is often the case with natural products. The recent advancement of first-principle-based computational chemistry techniques has provided an alternate method for obtaining precise BDE for any molecule of interest [12]. However, only at highly connected post Hartree–Fock levels of theory have *ab initio* approaches proven accurate among the known computational chemistry methods. The G2 and CCSD(T) approaches, for example, have been shown to be accurate but only for tiny compounds [13–19]. It is not feasible to perform all such calculations on supercomputers, and the above-stated advantages of DFT justify the less accurate but more practical method, especially for predicting the characteristics of very large molecules [5,10,11,19,20].

Seventeen density functionals stand out with their performance in calculating thermochemical parameters, listed in the electronic supplementary material, table S1, and thus used frequently for addressing such problems [1,21]. In particular, M05-2X, M05 and MPWB1 K are recommended for general thermochemistry, kinetics and non-covalent interactions involving nonmetals [1]. BB1 K, MPWB1 K, BMK and M05-2X gave the best result for barrier heights in the HTBH38/04 database [1]. M05-2X, B1B95, MPW1B95, BMK, M05, B98 and B97-2 gave a good performance for reaction energies in the HTBH38/04 database [1]. M05-2X, BMK, BB1 K, MPWB1 K and M05 were the best for kinetics in the HTBH38/04 database [1]. LC−*ω*PBE, M06-2X, BMK, B2PLYP, M05-2X and MN12SX yielded high-quality results in the calculation of rate constants for radical-molecule reactions in an aqueous solution [1]. M06 yields good results for transition-metal chemistry and other multi-reference cases [21]. The M08-HX functional performs better than M06-2X, M05-2X and B3LYP for a range of problems, including main-group thermochemistry, kinetics, non-covalent interactions and electronic spectroscopy [22].

Many previous studies demonstrated that the radical scavenging activity of aromatic compounds such as phenolics [23–29], aromatic amines [30–32] or thiols [33,34], particularly in lipid media, follows the hydrogen transfer mechanism characterized by BDE(X–H, X=C, N, O, S) values. While several studies addressed the optimization of model chemistries for calculating specific physico-chemical parameters of specific compound families [35–45], these works also highlight that the performance of model chemistries cannot be evaluated in the absolute and has to be repeated for each practical problem sufficiently different from the ones addressed before. One such problem is the computation of BDE(X–H, X=C, N, O, S) of compounds containing aromatic rings. Thus, in this study, the 17 DFT functionals identified above have been evaluated for computing BDE values of the X−H (X=C, N, O, S) bond of ArX−H compounds to find the most convenient method for computing this thermochemical parameter for this specific case.

## 2. Computational details

The Gaussian 16 package of programs was used for all calculations in our work. BDE calculations were performed according to the protocol of Wright [11] that is widely used in the literature [10,18–20,46]. In this study, the geometry is optimized, and zero-point energy corrections are calculated with the same model chemistry for all compounds and for all of the following by single-point calculations of BDE values with a range of model chemistries. The calculated BDEs were compared with the experimental BDE values obtained from the literature [47].

BDE is the total energy required for homolytic breakage of a specific bond, here the X−H of the ArX−H compound. BDE is thus the enthalpy change of the reaction

2.1 $$\mathrm{ArX}-H\;\to {\mathrm{ArX}}^{∙}+H^{∙}$$

expressed numerically as

2.2 $$\mathrm{BDE}=H({\mathrm{ArX}}^{∙})\;+\;H(H^{∙})\;-\;H(\mathrm{ArX}-H).$$

For the compounds that have multiple conformers, all of these were screened by the Spartan Software with the MMFF/molecular mechanics module [48] and the conformer with the lowest electronic energy was included in the analysis. ArX−H (X=C, N, O, S) substances were chosen as model compounds for performance testing of the 17 density functionals on the condition that experimental values of BDEs exist for them.

## 3. Results and discussion

### 3.1. Effect of the size of the basis set

Previous studies showed that the B3LYP functional is sufficient and accurate for geometry optimization and frequency calculations [20,21,49–52]. Thus, calculations were performed with B3LYP functional and five typical basis sets including 6-31G(d), 6-31 + G(d), 6-31 + G(d,p), 6-311G(d,p) and 6-311 ++ G(d,p)) for the geometry optimization and frequency calculations, followed by single-point calculations of BDEs using 17 different functionals and the 6-311 + G(3df,2p) level [21]. This is a protocol intended for modest computational resources, where the larger basis set is only used where it is necessary; the aim here is to validate this approach against experimental data.

In the first section, we assess the variation affected by the size of the basis sets by cross-comparison of the data. Arguably any improvement in the numerical values should manifest as a difference between the values calculated with different basis sets, and therefore the analysis does not require referencing experimental data to reveal the ideal size of the basis set. The detailed data including differences in numerical values between basis sets in comparison to the smallest 6-31G(d) level are presented in the electronic supplementary material, table S3–S6 for C−H, S8, S10 and S12 for N−H, O−H and S−H bonds, respectively. Given the very small variation observed for C−H bonds, for N−H, O−H and S−H bonds only the smallest and largest studied basis sets were compared.

As per these data, variations of the BDE values based on geometry optimization with different size basis sets are mostly lower than 0.3 kcal mol^−1^ (corresponding to 0.3%). The only exception was observed for BDE calculated with B2PLYP functional at the N−H bonds (electronic supplementary material, table S8), and the S−H bond of compounds SH1 and SH3 (electronic supplementary material, table S12). The larger error here is likely related to the chemical environment of these bonds that requires more accurate geometry optimization and is beyond the scope of this work to analyse in detail. Overall it was found that the geometry optimization with larger basis sets does not yield significant differences compared to smaller basis sets; hence even without comparison to experimental data, it is clear that they cannot deliver much improvement of accuracy (range from 0.1% to 0.3%). Importantly the data does not suggest, per se, that the size of the basis set in geometry optimization is irrelevant for BDE calculations, rather than the larger basis set is not expected to deliver substantial improvement, conversely for very large molecules reducing the size of the basis set is a viable compromise to obtain sensible predictions of BDE. As the few specific cases mentioned above imply, for higher accuracy, it is still desirable to perform calculations with a larger basis set. Therefore, in the next section, we focus our analysis on the geometry optimized with the smallest basis set, i.e. the DFT/6-311 + G(3df,2p)//B3LYP/6-31G(d) chemistry model, to evaluate the accuracy of different functionals in the calculation of BDE values of the studied compounds against experimental data.

### 3.2. The bond dissociation energy of C−H

In this part, the performance of 17 popular functionals was examined for computing the BDE values of 11 ArC−H substances, where hydrogen atoms are either directly bound to the aromatic ring or bound to a carbon atom that is a substituent on the aromatic ring. The compounds were benzene (CH1) [53], toluene (CH2) [54], ethylbenzene (CH3) [55], diphenylmethane (CH4) [56], indene (CH5) [57], tetralin (CH6) [58], 9-10-dihydroanthracene (CH8) [59], xanthene (CH9) [60], fluorine (CH10) [61] and 9-anthracenylmethane (CH11) [56]. All of these substances have accurate experimentally defined BDE values in the literature (electronic supplementary material, table S2) [47] that were used as a reference to assess the performance of the functionals.

As shown in table 1, the best-performing functionals for calculating BDE values of C−H substances are M08-HX, M06-2X, BMK and M05-2X, in that order. Remarkably, the mean unsigned errors (MUE) of M06-2X and BMK are equal to M08-HX (1.3 kcal mol^−1^). The maximum absolute error (MaxAE) of M08-HX has its minimum at 3.0 kcal mol^−1^. The MaxAE values of BMK and M05-2X are higher than those of the M08-HX and M06HX by about 1.0−1.6 kcal mol^−1^. A similar trend was observed at absolute values; the relative MUE of the four listed functionals above are not significantly different (range from 1.4% to 1.6%), whereas the relative MaxAE of BMK and M05-2X (4.8% and 5.5%, respectively) are much higher than that of M08-HX and M06-2X (3.9% and 3.8%, respectively). The rest of the tested functionals for the C−H bond BDE calculations also have the MaxAE lower than 10.0 kcal mol^−1^ except B2PLYP (12.0 kcal mol^−1^).

**Table 1.** Absolute (in kcal mol^−1^)^a^ and relative (in %) deviation of C−H BDEs from experimental values.

C−H BDE	CH1	CH2	CH3	CH4	CH5	CH6	CH7	CH8	CH9	CH 10	CH 11	absolute values		relative values^c^	
												MUE^b^	MaxAE^b^	MUE^b^	MaxAE^b^
M06-2X	−1.9	1.3	1.6	−1.4	−3.2	0.6	−0.7	2.5	0.2	0.0	−0.8	1.3	3.2	1.5	3.8
M05-2X	−0.4	−0.1	0.6	−2.4	−4.6	−0.5	0.9	1.3	−0.8	−1.1	−2.3	1.4	4.6	1.6	5.5
M06	−3.7	−1.4	−1.6	−5.2	−6.2	−2.9	−2.3	−1.6	−3.4	−3.5	−3.7	3.2	6.2	3.7	7.4
M05	−4.0	−3.1	−3.2	−7.0	−8.0	−4.8	−2.9	−3.2	−4.5	−5.0	−5.8	4.7	8.0	5.3	9.7
BMK	−1.0	0.5	0.9	−2.9	−4.0	−0.3	0.1	0.9	−1.3	−1.0	−1.7	*1.3*	*4.0*	*1.5*	*4.8*
MPW1B95	−1.7	−0.4	−0.6	−4.6	−5.5	−1.5	−0.6	−0.4	−3.0	−2.7	−2.6	2.2	5.5	2.5	6.7
B1B95	−2.4	−0.9	−1.2	−5.2	−6.1	−2.1	−1.3	−1.1	−3.7	−3.3	−3.1	2.8	6.1	3.1	7.4
B98	−2.7	−2.1	−1.8	−6.0	−6.7	−3.1	−1.8	−2.3	−4.4	−4.0	−4.0	3.5	6.7	4.0	8.1
B97-2	−3.1	−2.4	−2.4	−6.5	−7.5	−3.9	−2.1	−3.0	−5.2	−4.8	−4.6	4.1	7.5	4.7	9.1
LC-ωPBE	−4.3	−2.2	−1.9	−4.0	−5.3	−3.2	−3.2	−0.4	−2.1	−2.0	−6.3	3.2	6.3	3.6	7.5
B3LYP	−2.4	−2.1	−1.9	−6.1	−6.7	−3.3	−1.5	−2.5	−4.5	−4.1	−4.1	3.6	6.7	4.1	8.1
cam-B3LYP	−1.4	−0.8	−0.4	−3.7	−4.8	−1.8	−0.4	0.0	−1.9	−1.6	−3.5	1.8	4.8	2.1	5.7
B2PLYP	−8.9	−8.9	−7.9	−11.4	−11.9	−9.6	−8.0	−8.0	−9.1	−9.1	−12.0	9.5	12.0	10.8	14.3
MPWB1 K	−1.3	−0.1	−0.1	−3.7	−4.9	−1.1	−0.1	0.4	−1.9	−1.8	−2.4	1.6	4.9	1.8	5.9
BB1 K	−1.7	−0.4	−0.5	−4.2	−5.3	−1.5	−0.6	−0.1	−2.4	−2.2	−2.7	2.0	5.3	2.2	6.4
BB95	−4.0	−2.7	−3.3	−8.3	−8.3	−4.2	−3.1	−4.1	−7.2	−6.3	−5.1	5.1	8.3	5.9	9.9
M08-HX	−0.3	1.6	1.7	−1.1	−2.7	1.1	1.0	3.0	0.5	0.5	−0.4	1.3	3.0	1.4	3.9

^a^All tested substance geometries were pre-optimized with B3LYP/6-31G(d) functional, then were calculated single-point energy using 6-311 + G(3df,2p) basis set.

^b^MUE (mean unsigned error) = mean absolute deviation; MaxAE = max absolute error.

^c^The relative values of MUEs were calculated from it, and the average of BDE values of all C−H substances; the relative values of MaxAE were calculated from it and its corresponding value for BDE of C−H substances.

These results correlate well with reports on comparing the accuracy of various functionals for energetic calculations on specific classes of compounds. Yan Zhao *et al*. [21] tested nine functionals against three experimental databases of Izgorodina *et al*. [62], and found that M06-2X and M05-2X showed excellent performance for calculating energetics involving radicals as well as the alkyl oxygen BDEs of nitroxides. Similar results were also reported in other studies [38–42], once again asserting the superior performance of M06-2X and M05-2X over any other density functionals.

### 3.3. The bond dissociation energy of N−H

Nine ArN−H substances, which have the hydrogen atom directly bound to the aromatic ring or linked to the aromatic ring by a substituent nitrogen atom, were used to evaluate computing methods for N−H bond dissociation energies. The compounds were 4-flouroaniline (NH1) [63], *N*-methyl-*N*-phenylamine (NH2) [64], phenylhydrazine (NH3) [65], diphenylamine (NH4) [66], 1,3-oxazolidin-2-one (NH5) [67], 2-piperidone (NH6) [68], 2,2,4-trimethyl-1,2,3,4-tetrahydroquinoline (NH7) [69], carbazole (NH8) [70] and dimethyl-phenothioazine (NH9) [71]. The experimental BDE values were collected from the literature (electronic supplementary material, table S7) [47].

The calculated results (table 2) show that M05-2X and M06-2X are again the best performers with minor deviations, MUEs being 1.0 kcal mol^−1^ and 1.2 kcal mol^−1^, respectively. Their MaxAEs are minor, with 2.6 kcal mol^−1^ for M05-2X and 2.9 kcal mol^−1^ for M06-2X. These values are lower than in the case of the C−H bond (4.6 kcal mol^−1^ for M05-2X and 3.2 kcal mol^−1^ for M06-2X). The M08-HX and MPWB1 K functionals have an equal error of 1.8 kcal mol^−1^ of the absolute MUE, whereas the relative MUEs of M05-2X, M06-2X, M08-HX and MPWB1 K were 1.1% to 2.0%. The BMK functional yields a good MUE with 1.9 kcal mol^−1^, and MaxAE is 4.0 kcal mol^−1^; however, the MUEs of the rest of the density functionals range from 2.1 kcal mol^−1^ to 6.1 kcal mol^−1^.

**Table 2.** Absolute (in kcal mol^−1^)^a^ and relative (in %) deviation of N−H BDEs from experimental values.

N-H BDE	NH1	NH2	NH3	NH4	NH5	NH6	NH7	NH8	NH9	absolute values		relative values^c^	
										MUE^b^	MaxAE^b^	MUE^b^	MaxAE^b^
M06-2X	2.6	1.0	2.6	0.1	−0.4	−2.9	1.1	−0.2	0.1	1.2	2.9	1.4	2.6
M05-2X	1.9	0.1	1.9	−0.8	−0.1	−2.6	0.2	−0.8	−0.3	1.0	2.6	1.1	2.4
M06	−0.8	−2.5	0.2	−3.8	−2.5	−4.9	−2.4	−4.6	−4.1	2.9	4.9	3.2	4.5
M05	−2.3	−4.0	−1.4	−5.1	−3.6	−5.7	−4.6	−5.6	−5.1	4.1	5.7	4.6	5.2
BMK	0.5	−1.3	0.6	−2.4	−1.8	−4.0	−1.7	−2.5	−2.1	1.9	4.0	2.1	3.7
MPW1B95	0.2	−1.8	−0.3	−3.1	−2.5	−4.7	−1.9	−3.3	−3.0	2.3	4.7	2.5	4.3
B1B95	−0.5	−2.4	−1.1	−3.8	−3.3	−5.5	−2.7	−4.0	−3.9	3.0	5.5	3.3	5.0
B98	−2.0	−3.9	−1.6	−5.5	−4.9	−6.9	−4.4	−6.1	−5.4	4.5	6.9	5.0	6.3
B97-2	−2.2	−4.2	−2.4	−5.8	−5.1	−7.1	−4.7	−6.2	−6.3	4.9	7.1	5.4	6.5
LC-wPBE	−1.0	−2.8	−0.3	−2.5	−3.0	−5.4	−3.0	−2.4	−1.4	2.4	5.4	2.7	5.0
B3LYP	−1.8	−3.8	−1.6	−5.3	−4.7	−6.7	−4.3	−5.9	−5.4	4.4	6.7	4.9	6.1
cam-B3LYP	−0.2	−2.0	0.4	−2.7	−2.5	−4.8	−2.4	−3.0	−2.1	2.2	4.8	2.5	4.3
B2PLYP	−2.2	−3.7	0.2	−4.7	−2.1	−5.2	−4.4	−5.0	−3.5	3.5	5.2	3.8	4.7
MPWB1 K	0.6	−1.3	0.7	−2.2	−1.5	−4.1	−1.4	−2.3	−1.8	*1.8*	*4.1*	*2.0*	*3.7*
BB1 K	0.2	−1.8	0.2	−2.8	−2.1	−4.6	−1.9	−2.9	−2.5	2.1	4.6	2.3	4.2
BB95	−2.8	−4.8	−4.5	−7.2	−6.8	−8.4	−5.3	−7.6	−7.6	6.1	8.4	6.8	7.6
M08-HX	3.7	1.8	3.1	1.1	0.5	−1.4	2.1	1.3	1.1	1.8	3.7	2.0	4.1

^a^All tested substance geometries were pre-optimized with B3LYP/6-31G(d) functional, then were calculated single-point energy using 6-311 + G(3df,2p) basis set.

^b^MUE (mean unsigned error) = mean absolute deviation; MaxAE = max absolute error.

^c^The relative values of MUEs were calculated from it and the average of BDE values of all N−H substances; the relative values of MaxAE were calculated from it and its corresponding value for BDE of N−H substances.

### 3.4. The bond dissociation energy of O−H

BDEs of ArO−H substances were calculated here for 4-chlorophenol (OH1) [72], 2-methoxyphenol (OH2) [73], 4-methoxy-2,3,6-trimethylphenol (OH3) [74], 2,2,5,7,8-pentamethyl-chroman-6-ol (OH4) [74], 2,3,5-trimethyl-chroman-4-ol (OH5) [69], ubiquinol-2 (OH6) [69], 2,2,6,7-tetramethyl-2,3-dihydrobenzofuran-5-ol (OH7) [69], 1-hydroxynaphthalene (OH8) [75], 4,4′-dihydroxybiphenyl (OH9) [75] and 1-hydroxy-phenanthrene (OH10) [69] that all have accurate experimental BDE values in the literature [47] (electronic supplementary material, table S9). The comparison of density functionals is presented in table 3.

**Table 3.** Absolute (in kcal mol^−1^)^a^ and relative (in %) deviation of O−H BDEs from experimental values.

O-H BDE	OH1	OH2	OH3	OH4	OH5	OH6	OH7	OH8	OH9	OH10	absolute values		relative values^c^	
											MUE^b^	MaxAE^b^	MUE^b^	MaxAE^b^
M06-2X	−2.2	1.8	0.0	−0.1	−0.7	3.1	0.3	−1.7	1.2	−0.6	1.2	3.3	1.4	4.0
M05-2X	−2.6	1.8	−0.4	−0.5	−1.1	2.9	0.0	−2.8	0.8	−1.4	1.4	3.2	1.7	3.9
M06	−6.8	−2.4	−4.2	−4.6	−5.1	−0.7	−4.0	−6.4	−3.8	−5.4	4.2	6.7	5.1	7.4
M05	−8.8	−4.9	−6.3	−6.8	−7.3	−3.2	−6.3	−9.1	−5.7	−8.1	6.6	9.1	7.9	10.8
BMK	−5.7	−2.0	−3.9	−4.2	−4.7	−0.9	−3.8	−5.5	−2.7	−4.5	3.7	5.7	4.4	6.3
MPW1B95	−5.4	−1.1	−3.0	−3.3	−3.8	−0.3	−2.8	−4.7	−2.3	−3.9	3.0	5.4	3.6	5.9
B1B95	−6.2	−2.0	−3.8	−4.1	−4.7	−1.3	−3.6	−5.5	−3.1	−4.7	3.8	6.1	4.6	6.8
B98	−7.9	−4.0	−5.7	−6.2	−6.6	−3.2	−5.7	−7.5	−5.0	−6.7	5.8	7.9	7.0	8.8
B97-2	−8.4	−4.5	−6.2	−6.7	−7.2	−3.9	−6.1	−7.9	−5.2	−7.1	6.3	8.3	7.5	9.2
LC-ωPBE	−7.4	−3.1	−4.8	−5.0	−5.6	−1.5	−4.3	−7.9	−3.5	−6.5	4.8	7.8	5.9	9.3
B3LYP	−7.8	−3.7	−5.5	−6.0	−6.5	−2.5	−5.5	−7.3	−4.9	−6.6	5.6	7.9	6.8	8.7
cam-B3LYP	−6.0	−1.5	−3.6	−3.9	−4.4	0.0	−3.4	−5.8	−2.6	−4.7	3.6	5.9	4.3	6.6
B2PLYP	−14.3	−9.3	−11.0	−11.3	−11.8	−6.7	−10.7	−14.0	−10.8	−13.0	11.3	14.4	13.6	15.9
MPWB1 K	−4.6	0.0	−2.0	−2.2	−2.8	1.1	−1.7	−4.0	−1.4	−3.1	2.3	4.6	2.7	5.1
BB1 K	−5.2	−0.6	−2.6	−2.8	−3.4	0.4	−2.3	−4.6	−1.9	−3.6	2.7	5.1	3.2	5.7
BB95	−9.4	−5.8	−7.4	−8.0	−8.5	−5.9	−7.5	−8.4	−7.0	−8.1	7.5	9.4	9.1	10.4
M08-HX	−1.6	2.2	0.6	0.4	−0.1	3.2	0.8	−1.2	1.7	0.0	1.3	3.4	1.5	4.1

^a^All tested substance geometries were pre-optimized with B3LYP/6-31G(d) functional, then were calculated single-point energy using 6-311 + G(3df,2p) basis set.

^b^MUE (mean unsigned error) = mean absolute deviation; MaxAE = max absolute error.

^c^The relative values of MUEs were calculated from it and the average of BDE values of all O−H substances; the relative values of MaxAE were calculated from it and its corresponding value for BDE of O−H substance.

M06-2X, M05-2X and M08-HX functionals give the best performance for O−H BDE calculations. All three functionals have similar MUE and Max AE in both absolute as well as relative values. MUEs of the three functionals above range from 1.2 kcal mol^−1^ to 1.4 kcal mol^−1^ (corresponding to 1.4% to 1.7% in relative value) and MaxAEs range from 3.2 kcal mol^−1^ to 3.4 kcal mol^−1^ (corresponding to 3.9% to 4.1% in relative value). Based on the computed data, BMK and MPWB1 K are not as good as in the N−H BDE calculations. BMK has MUE and MaxAE of 3.7 kcal mol^−1^ and 5.7 kcal mol^−1^, respectively, while those of MPWB1 K are 2.3 kcal mol^−1^ for MUE and 4.6 kcal mol^−1^ for MaxAE. B2PLYP is also not good for N−H BDE calculations, with MUE = 11.3 kcal mol^−1^. Thus, this suggests that BMK, MPWB1 K and B2PLYP should not be used to compute the BDE value of the N−H bond.

### 3.5. The bond dissociation energy of S−H

For the S−H bond calculations, nine ArS−H substances were selected from the literature (electronic supplementary material, table S11) [47]: benzenethiol (SH1) [69], 2-methyl-benzenethiol (SH2) [76], 3-trifluoromethyl-benzenethiol (SH3) [69], 4-amino-benzenethiol (SH4) [76], 1-naphthalenethiol (SH5) [69], 4-chloro-benzenethiol (SH6) [76], 4-methyl-benzenethiol (SH7) [76], 4-methoxy-benzenethiol (SH8) [76] and 4-ethoxy-benzenethiol (SH9) [69]. The results are presented in table 4.

**Table 4.** Absolute (in kcal mol^−1^)^a^ and relative (in %) deviation of S−H BDEs from reference values.

S-H BDE	SH1	SH2	SH3	SH4	SH5	SH6	SH7	SH8	SH9	absolute values		relative values^c^	
										MUE^b^	MaxAE^b^	MUE^b^	MaxAE^b^
M06-2X	−0.8	0.7	0.0	5.4	−0.4	0.2	0.5	−0.1	−2.1	1.1	5.4	1.5	7.8
M05-2X	−2.1	−0.4	−1.2	4.5	−1.7	−1.0	−0.8	−1.0	−3.0	1.8	4.5	2.3	6.5
M06	−2.5	−1.5	−1.6	3.1	−2.8	−1.7	−1.4	−2.3	−4.3	2.3	4.3	3.0	5.4
M05	−3.3	−2.3	−2.4	2.5	−3.9	−2.3	−2.1	−3.2	−5.2	3.0	5.2	3.9	6.6
BMK	−2.6	−1.1	−1.7	3.4	−2.5	−1.6	−1.4	−2.0	−4.0	2.3	4.0	2.9	5.0
MPW1B95	7.6	−0.6	7.6	3.6	−2.1	−1.1	−0.8	−1.7	−3.7	3.2	7.6	4.1	9.4
B1B95	7.1	−1.2	7.1	3.0	−2.7	−1.6	−1.3	−2.3	−4.3	3.4	7.1	4.3	8.8
B98	−4.1	−2.8	−3.1	1.8	−4.3	−3.1	−2.9	−3.6	−5.6	3.5	5.6	4.5	7.1
B97-2	−4.1	−2.9	−3.1	1.6	−4.4	−3.2	−2.9	−3.8	−5.9	3.5	5.9	4.5	7.4
LC-wPBE	−3.5	−2.0	−2.8	3.7	−3.4	−2.3	−2.0	−2.1	−4.0	2.9	4.0	3.7	5.1
B3LYP	6.0	−2.5	−2.7	2.0	−3.9	−2.8	−2.6	−3.3	−5.4	3.5	6.0	4.5	7.4
cam-B3LYP	−2.7	−1.2	−1.8	4.0	−2.5	−1.5	−1.4	−1.6	−3.6	2.2	4.0	2.9	5.8
B2PLYP	−1.2	−9.0	−9.9	-7.3	−10.5	−9.4	−9.2	−8.8	−10.8	8.5	10.8	10.8	13.7
MPWB1 K	7.6	−0.3	7.6	4.4	−1.8	−0.8	−0.6	−1.1	−3.1	3.0	7.6	3.9	9.5
BB1 K	7.2	−0.7	7.3	4.0	−2.2	−1.2	−0.9	−1.5	−3.5	3.1	7.3	4.0	9.0
BB95	−3.5	−2.6	−2.3	0.5	−4.4	−3.1	−2.6	−4.6	−6.6	3.4	6.6	4.3	8.4
M08-HX	0.1	1.6	1.0	6.2	0.5	1.1	1.4	0.9	−1.1	1.6	6.2	2.0	8.9

^a^All tested substance geometries were pre-optimized with B3LYP/6-31G(d) functional, then were calculated single-point energy using 6-311 + G(3df,2p) basis set.

^b^MUE (mean unsigned error) = mean absolute deviation; MaxAE = max absolute error.

^c^The relative values of MUEs were calculated from it and the average of BDE values of all O−H substances; the relative values of MaxAE were calculated from it and its corresponding value for BDE of O−H substances.

The results show that M06-2X is the best density functional for this dataset. Its MUE and MaxAE are 1.1 kcal mol^−1^ and 5.4 kcal mol^−1^, respectively. At the same time, M05-2X and M08-HX also have good accuracy with 1.8 kcal mol^−1^ and 1.6 kcal mol^−1^. However, the MaxAE (both absolute and relative values) of those functionals are higher than those of C−H BDEs, N−H BDEs and O−H BDEs calculations, which are in the range of 4.5 kcal mol^−1^ to 6.2 kcal mol^−1^. The rest of the tested functionals have similar deviations ranging from 2.3 kcal mol^−1^ to 3.5 kcal mol^−1^, except the B2PLYP functional with 8.5 kcal mol^−1^ of absolute MUE. Thus BDEs of the S−H bond could be calculated effectively by the Minnesota functionals.

### 3.6. Average deviation

To evaluate the accuracy and effectiveness of the computational methods, the average of MUE and MaxAE of each functional were compared for an overall assessment of their performance in calculating the BDE values. The results are presented in table 5.

**Table 5.** The average absolute (in kcal mol^−1^) and relative (in %) deviation of BDE values from the experimental reference values.

functionals	absolute value		relative value	
	MUE	MaxAE	MUE	MaxAE
M06-2X	1.2	3.7	1.4	4.5
M05-2X	1.4	3.7	1.6	4.6
M06	3.2	5.5	3.7	6.2
M05	4.6	7.0	5.4	8.1
BMK	2.3	4.4	2.7	5.0
MPW1B95	2.7	5.8	3.2	6.6
B1B95	3.2	6.2	3.9	7.0
B98	4.3	6.8	5.1	7.6
B97-2	4.7	7.2	5.6	8.1
LC-ωPBE	3.3	5.9	3.9	6.7
B3LYP	4.3	6.8	5.0	7.6
cam-B3LYP	2.5	4.9	2.9	5.6
B2PLYP	8.2	10.6	9.8	12.2
MPWB1 K	2.2	5.3	2.6	6.0
BB1 K	2.5	5.6	3.0	6.3
BB95	5.5	8.2	6.5	9.1
M08-HX	1.5	4.1	1.7	5.3

M06-2X, M05-2X and M08-HX yield higher accuracy than any other tested functionals. M06-2X is the best performer, with the lowest absolute and relative value deviation. M05-2X and M08-HX have good accuracy; however, the MUE and MaxAE values are slightly higher than those of M06-2X. All of the other functionals have higher errors in BDEs compared to the Minnesota functionals. Therefore, based on computed data, the M06-2X, M05-2X and M08-HX functionals offer the most effective and accurate methods to compute BDE values of ArX−H (X=C, N, O, S) bonds.

## 4. Conclusion

In this study, the BDE(X−H, X=C, N, O, S) values of typical aromatic compounds have been computed and compared using 17 different DFT functionals, based on the protocol by Wright [11]. The results showed that M06-2X, M05-2X and M08-HX yield higher accuracy BDE values than any other tested functionals. The 6-31G(d) basis set with the B3LYP functional is sufficient for geometry optimization, with acceptable absolute and relative value deviation, whereas the larger basis sets 6-31 + G(d), 6-31 + G(d,p), 6-311G(d,p) and 6-311 ++ G(d,p) did not deliver a significant difference. Hence, it is possible to obtain reasonable predictions of BDE of very large molecules based on geometry optimization with a small basis set without substantial loss of accuracy. It should be emphasized that this does not apply to the single-point BDE calculation, where a large basis set was used for accurate results. When compared to experimental data, the M06-2X, M05-2X and M08-HX density functionals were found to offer the most affordable performance for calculating the BDEs of ArX−H (X=C, N, O, S) bonds.

## Data Availability

All relevant necessary data to reproduce all results in the paper are within the main text, electronic supplementary material (https://doi.org/10.5281/zenodo.6052741) and the Dryad Digital Repository: https://doi.org/10.5061/dryad.1c59zw3x3 [77].
